# Effect of Tannic Acid Concentrations on Temperature-Sensitive Sol–Gel Transition and Stability of Tannic Acid/Pluronic F127 Composite Hydrogels

**DOI:** 10.3390/gels10040256

**Published:** 2024-04-10

**Authors:** Jeong Yun Lee, Hyun Ho Shin, Chungyeon Cho, Ji Hyun Ryu

**Affiliations:** 1 Department of Carbon Convergence Engineering, Wonkwang University, Iksan 54538, Jeonbuk, Republic of Korea; wjddbs4058@wku.ac.kr; 2 Department of Chemical Engineering, Wonkwang University, Iksan 54538, Jeonbuk, Republic of Korea; tlsgusgh1231@wku.ac.kr; 3 Smart Convergence Materials Analysis Center, Wonkwang University, Iksan 54538, Jeonbuk, Republic of Korea

**Keywords:** tannic acid, Pluronic, temperature-sensitive, hydrogen bond, adhesive, bursting pressure

## Abstract

Recently, interest in polyphenol-containing composite adhesives for various biomedical applications has been growing. Tannic acid (TA) is a polyphenolic compound with advantageous properties, including antioxidant and antimicrobial properties. Additionally, TA contains multiple hydroxyl groups that exhibit biological activity by forming hydrogen bonds with proteins and biomacromolecules. Furthermore, TA-containing polymer composites exhibit excellent tissue adhesion properties. In this study, the gelation behavior and adhesion forces of TA/Pluronic F127 (TA/PluF) composite hydrogels were investigated by varying the TA and PluF concentrations. PluF (above 16 wt%) alone showed temperature-responsive gelation behavior because of the closely packed micelle aggregates. After the addition of a small amount of TA, the TA/PluF hydrogels showed thermosensitive behavior similar to that of PluF hydrogels. However, the TA/PluF hydrogels containing more than 10 wt% TA completely suppressed the thermo-responsive gelation kinetics of PluF, which may have been due to the hydrogen bonds between TA and PluF. In addition, TA/PluF hydrogels with 40 wt% TA showed excellent tissue adhesion properties and bursting pressure in porcine intestinal tissues. These results are expected to aid in understanding the use of mixtures of TA and thermosensitive block copolymers to fabricate adhesive hydrogels for versatile biomedical applications.

## 1. Introduction

Polyphenols are a diverse group of naturally occurring compounds that are widely recognized for their presence in various plants [1]. Polyphenols are generally categorized into flavonoids (i.e., catechin, epicatechin, quercetin, and malvidin-3-glucoside), phenolic acids (hydroxycinnamic acid and hydroxybenzoic acid), lignans, and stilbenes, each differing in their core structural elements and biological functions [1,2,3,4]. Their structural differences determine their role in plants, as well as influence their bioavailability, metabolism, and biological effects in humans [4,5,6]. Generally, polyphenols are renowned for their rich hydroxyl groups attached to aromatic rings, which fundamentally contribute to their chemical properties and biological activities [4,5,6]. For instance, polyphenols can scavenge free radicals, thereby decreasing oxidative stress, a known contributor to various chronic diseases [4,5,6]. Additionally, the antioxidant, anti-inflammatory, and anticarcinogenic properties of polyphenols enable a wide range of biomedical applications [5,6,7,8]. Therefore, the development of polyphenol-based materials for therapeutic applications is highly desirable.

Tannic acid (TA) is a polyphenol with unique characteristics that render it valuable in various fields, including the food, beverage, cosmetic, and biomedical industries [9,10,11]. In addition to its antioxidant and antibacterial properties, TA has a strong binding capability with proteins, polysaccharides, and biomacromolecules via physical and chemical interactions (i.e., hydrogen bonds, coordinative bonds, electrostatic interactions, hydrophobic interactions, and oxidation-mediated polymerization) because of the abundance of multiple hydroxyl groups along its galloyl moieties [12,13,14,15,16]. For instance, TA can be used for protein precipitation or stabilization via hydrogen bonding and hydrophobic interactions with proteins [11,17,18]. Additionally, TA-based composite materials exhibit excellent tissue adhesive properties that can be applied in versatile biomedical applications, such as wound-healing, hemostatic, and tissue-sealing materials [14,19]. In addition, TA contributes to the functional properties (i.e., stretchable, adhesive, and self-healing properties) and biological functions (i.e., antioxidant and antimicrobial characteristics) of polymeric networks [20,21,22,23]. However, there are concerns regarding TA toxicity. The LD50 value of TA by oral administration is 2260 mg/kg body weight in rats, as previously reported [11,24]. At a TA dose of LD50, developments of necrosis, nephritis, and acute gastroenteritis occur, resulting in death [11,24,25]. Although TA exhibits significant toxicity at high concentrations, the therapeutic window of TA shows no significant toxicity [11]. Thus, the development of adhesive materials using TA is urgent but requires careful consideration of their concentration and formulation to maximize their benefits while minimizing potential risks.

Stimuli-sensitive materials have been widely developed for various biomedical applications, particularly for drug delivery and tissue engineering [26]. The properties or behaviors of responsive materials are significantly changed in response to external stimuli, such as temperature changes, pH changes, and light exposure [26]. Pluronic is a representative synthetic thermosensitive polymer composed of polyethylene oxide (PEO) and polypropylene oxide (PPO), typically represented as PEO-PPO-PEO [27,28]. Pluronic exhibits a sol-to-gel transition above the low critical solution temperature (LCST) owing to the formation of closely packed micelles [27,28,29]. Owing to these temperature-responsive properties, Pluronic is valuable in various pharmaceutical and biomedical applications, including drug delivery systems and tissue engineering [30,31,32]. One of the significant challenges in the use of Pluronic without modifications and additives is its erosion profile under physiological conditions, which can lead to its instability in biological environments [33]. Pluronic-based composite hydrogels have been synthesized to enhance its long-term stability. By incorporating polymers such as chitosan, hyaluronic acid, and alginate, the long-term stability of Pluronic-based composite hydrogels can be significantly enhanced [34,35,36,37]. For instance, terminally thiolated Pluronic F127 (Plu-SH) and catechol-conjugated chitosan (CHI-C) composite hydrogels exhibit enhanced stability in vitro and in vivo [37]. Additionally, the mass erosion rates of the CHI-C/Plu-SH hydrogels can be controlled from a few days to several months according to the Plu-SH concentration [37]. In addition, rapid hemostasis can be achieved using CHI-C/Plu-SH hydrogels because of their hemostatic capability and physical barrier formation [37,38]. Pluronic with these biopolymers has enhanced long-term stability while retaining the beneficial properties of each component. Therefore, the design of Pluronic-based materials is crucial for various pharmaceutical and biomedical applications.

TA and Pluronic composite materials have been developed for various biomedical applications. For instance, TA/Pluronic nanoparticles loaded with dexamethasone exhibit the stimuli-sensitive drug release profiles in the presence of esterase [39]. The nanoparticles also show the inflamed colon-targeting capability with reactive oxygen species (ROS) scavenging ability that is useful for the treatment of inflammatory bowel diseases [39]. In addition, curcumin-entrapped TA/Pluronic nano-assemblies show the enhanced cellular uptake of curcumin with bioactivity against breast cancer cells [40]. TA/Pluronic elastic gels exhibit excellent tissue adhesiveness with sealing effects [41]. In addition, the elastic gels significantly promote the neural function recovery of spinal cord injury [41]. In this study, the gelation behavior and in vitro stability of TA/Pluronic composite hydrogels with various TA concentrations were investigated to obtain a fundamental understanding of their physical and chemical characteristics. The temperature-sensitive sol–gel transition behavior and rheological properties of the TA/Pluronic hydrogels were examined using a rotational rheometer. In addition, the in vitro stability of the TA/Pluronic hydrogels in PBS solutions (pH 7.4) was monitored by determining the mass erosion rate over time. Furthermore, the in vitro tissue adhesion properties of the TA/Pluronic hydrogels with porcine intestinal tissues were quantitatively evaluated using a universal testing machine. The bursting pressures of the porcine intestine with a 1 cm long incision before and after applying the TA/Pluronic hydrogels were quantitatively measured using bursting pressure-monitoring devices composed of containers, indicators, pressure transmitters, and recorders. This work reveals how the viscoelastic properties of TA and Pluronic composites change as a function of TA concentration, offering significant insights for the development of TA-based adhesive materials in biomedical fields.

## 2. Results and Discussion

### 2.1. Effects of TA on Sol–Gel Curves of Tannic Acid/Pluronic (TA/PluF) Composite Hydrogels

**Figure 1:**
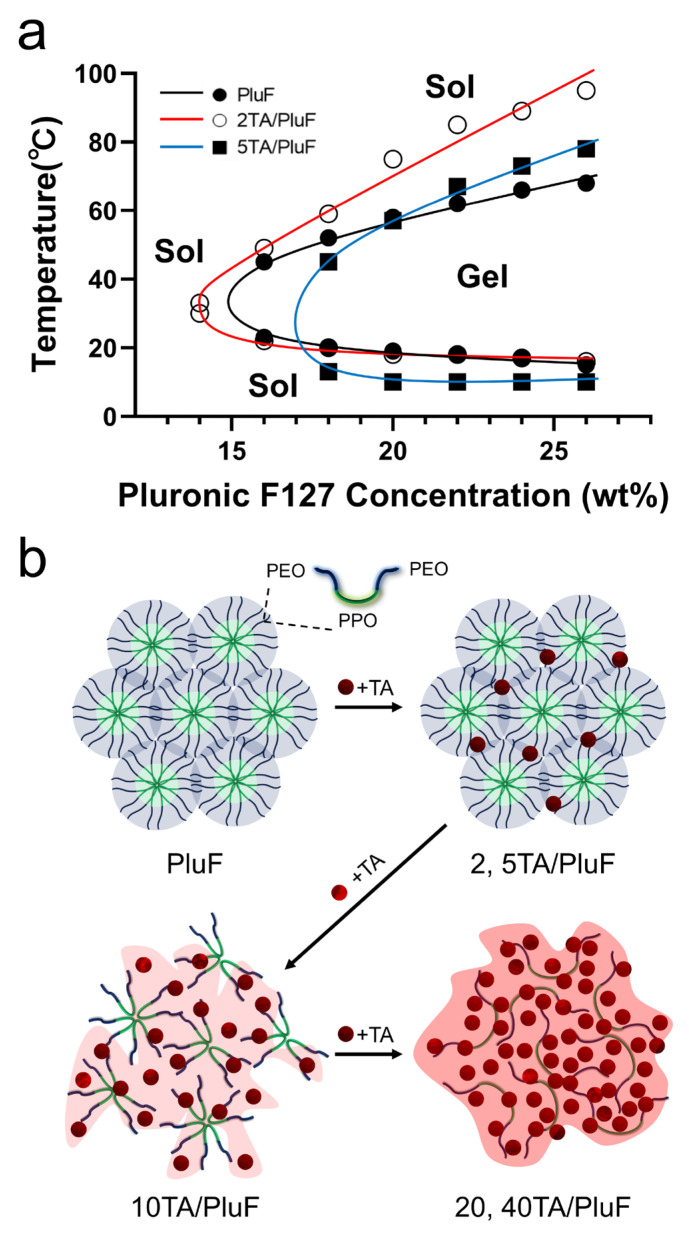
(**a**) Sol–gel transition curves of PluF (black line), 2TA/PluF (red line), and 5TA/PluF (blue line) hydrogels. (**b**) Schematic illustrations of TA/PluF hydrogel formation.

The TA molecules significantly affected the temperature-sensitive sol–gel transition behavior of the tannic acid/Pluronic (TA/PluF) composite hydrogels. To monitor the sol–gel transition of the TA/PluF hydrogels, vial-inversion tests were performed with various concentrations of TA and PluF. PluF alone showed gelation behavior at 16 wt% above the LCST (Figure 1a, black). As previously reported, PluF is a symmetric triblock copolymer composed of poly(ethylene oxide)–poly(propylene oxide)–poly(ethylene oxide) (PEO-PPO-PEO) that forms closely packed micelles above the LCST [42,43]. The LCSTs of PluF were 20, 19, 18, and 16 °C at 18, 20, 22, and 24 wt%, respectively. After the addition of TA at a final concentration of 2 wt%, the sol–gel curves of the 2TA/PluF hydrogels were similar to those of the PluF hydrogels (Figure 1a, red). The LCSTs of 2TA/PluF hydrogels were 30, 22, 20, 18, 18, and 17 °C at 14, 16, 18, 20, 22, and 24 wt% PluF, respectively. After the addition of TA at a final concentration of 5 wt%, the gelation temperatures of 5TA/PluF hydrogels were slightly decreased (13, 10, 10, and 10 °C for 18, 20, 22, and 24 wt% PluF, respectively), but no gelation was found in the 5TA/PluF hydrogels with 14 and 16 wt% PluF (Figure 1a, blue). More importantly, no significant sol–gel transition behavior was observed in TA/PluF hydrogels with TA concentrations above 10 wt% (10, 20, and 40TA/PluF hydrogels). Thus, the increase in the TA concentration in the TA/PluF composite hydrogels significantly hindered the temperature-responsive sol–gel transition behavior. Previous reports support our results that the intermolecular hydrogen bonds between TA and thermosensitive polymers (i.e., PluF and poly(N-isopropylacrylamide)) suppress temperature-responsive properties [41,44,45]. As illustrated in Figure 1b, the PluF hydrogels formed closely packed micelles above the LCST. However, the addition of TA to the TA/PluF hydrogels affected their temperature-responsive properties. The temperature-responsive properties remained at TA concentrations of 2 and 5 wt% in the TA/PluF hydrogels. However, it was difficult to clearly distinguish between the sol and gel states of 10TA/PluF hydrogels using the vial-inversion test. In addition, the temperature-responsive sol–gel transitions were not observed in the 20TA/PluF or 40TA/PluF hydrogels because of intermolecular hydrogen bonding between TA and PluF.

### 2.2. Rheological Properties of TA/PluF Hydrogels

**Figure 2:**
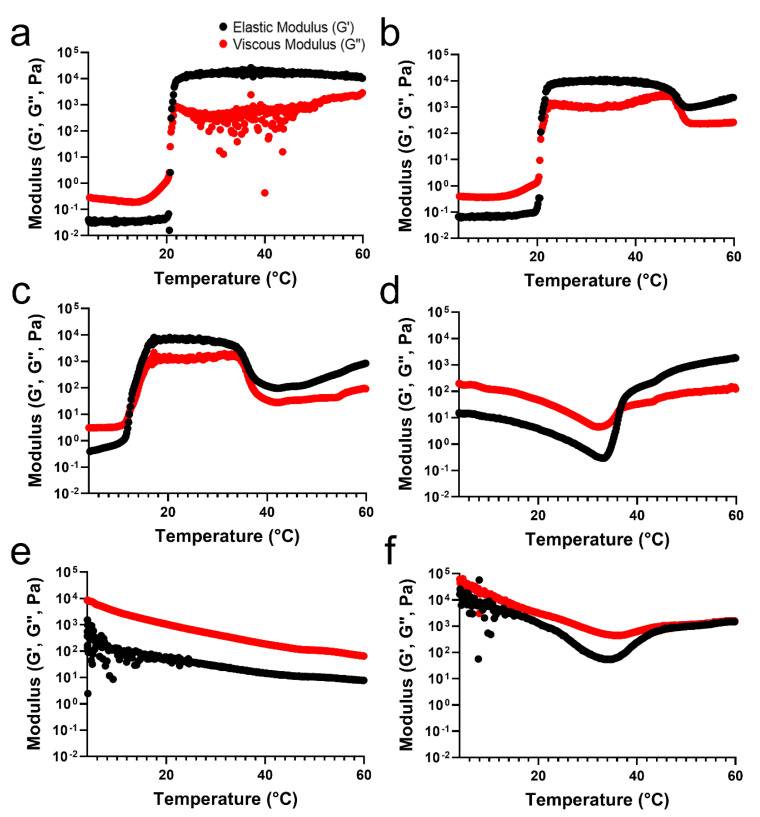
Temperature-responsive elastic (G′) and viscous (G″) modulus value changes in PluF (**a**), 2TA/PluF (**b**), 5TA/PluF (**c**), 10TA/PluF (**d**), 20TA/PluF (**e**), and 40TA/PluF (**f**).

To verify the differences in the temperature-sensitive properties of the TA/PluF hydrogels compared with those of the PluF hydrogels, a rheological analysis of TA/PluF hydrogels was performed using a rotational rheometer. For an adequate comparison to confirm the effects of the TA concentration on the temperature-sensitive properties, the final concentration of PluF in the TA/PluF hydrogels was fixed at 18 wt%. The crossover points of the elastic (G′) and viscous (G″) moduli were considered as the gelation temperature. Figure 2a presents the elastic (G′) and viscous (G″) modulus value changes in the PluF hydrogels as a function of temperature. The modulus (G′ and G″) values of PluF greatly increased at a gelation temperature of 20 °C, which were the crossover points of G′ and G”. To monitor the effects of TA addition, the final TA concentrations in the TA/PluF hydrogels were 2, 5, 10, 20, and 40 wt%. The gelation temperatures of TA/PluF hydrogels were reduced to 20 and 12 °C for 2 and 5 wt% TA, respectively (Figure 2b,c). Additionally, the slight increases in the G′ and G″ values of both 2TA/PluF and 5TA/PluF composite hydrogels were observed from 4 °C to room temperature. However, the TA/PluF composites with a TA concentration of 10 wt% showed different rheological properties from those of the PluF hydrogels. As shown in Figure 2d, both the G′ and G″ values of 10TA/PluF hydrogels were significantly higher than those of the PluF, 2TA/PluF, and 5TA/PluF hydrogels at 4 °C. In addition, both the G′ and G″ values decreased until 34 °C, and the crossover points of G′ and G″ occurred at approximately 36 °C. Furthermore, the G′ and G″ values further increased by increasing the TA concentrations from 10 to 40 wt% at 4 °C (Figure 2e,f). In addition, no crossover points for the 20TA/PluF and 40TA/PluF composites were found in the temperature sweep measurements.

**Figure 3:**
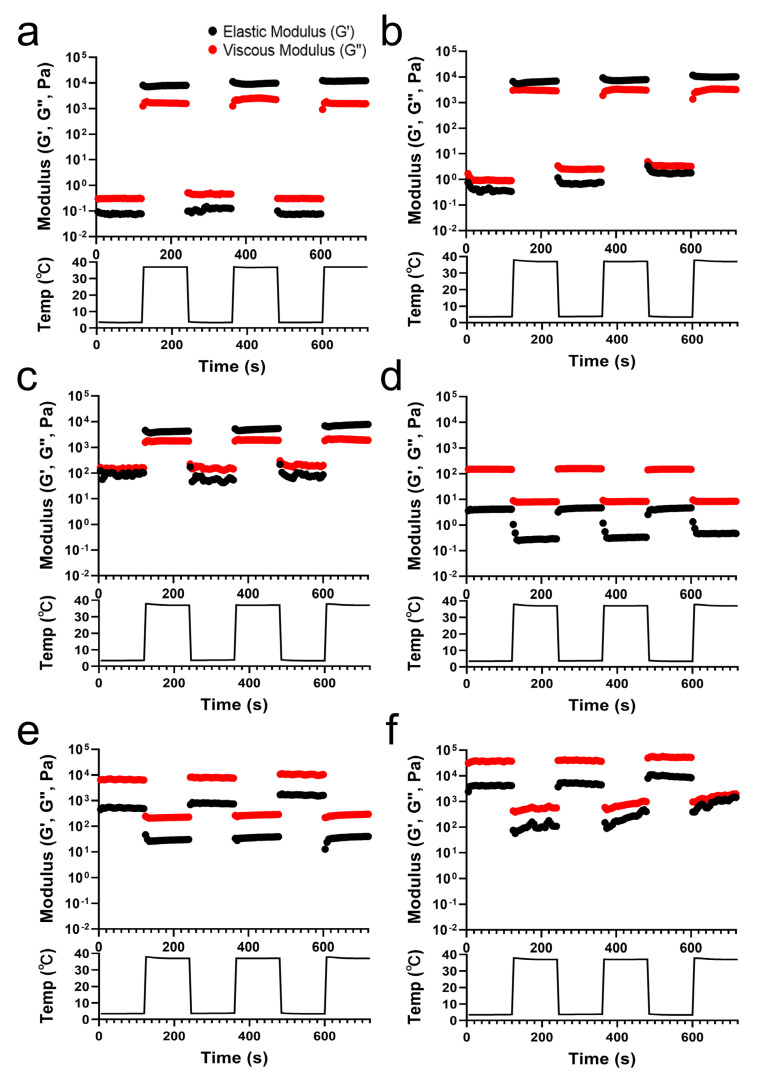
Elastic (G′) and viscous (G″) modulus changes in PluF (**a**), 2TA/PluF (**b**), 5TA/PluF (**c**), 10TA/PluF (**d**), 20TA/PluF (**e**), and 40TA/PluF (**f**) with temperature cycles of cooling (4 °C) and heating (37 °C).

The G′ and G″ values of the TA/PluF composites were monitored with temperature cycles of heating (37 °C) and cooling (4 °C) (Figure 3). PluF (Figure 3a), 2TA/PluF (Figure 3b), and 5TA/PluF (Figure 3c) hydrogels exhibited instant gelation at a physiological temperature (37 °C) and underwent the gel-to-sol transition at 4 °C. The hydrogels exhibited a reversible sol–gel transition at temperature cycles of 4 and 37 °C. It was noteworthy that the hydrogels could be stored at refrigerator temperatures (4 °C) and could form hydrogels under physiologically relevant conditions, indicating that they are highly suitable for biomedical applications. However, the G′ values were lower than the G″ values in the 10, 20, and 40TA/PluF composites at both 4 and 37 °C. In addition, both the G′ and G″ values in 10, 20, and 40TA/PluF decreased when changing the temperature from 4 to 37 °C due to the dissociation of hydrogen bonds between TA and PluF. As previously reported, the hydrogen bonds between phenolic compounds and PluF dissociate with increasing temperature [41]. Thus, the addition of TA to the TA/PluF composites significantly affected their temperature-responsive properties.

**Figure 4:**
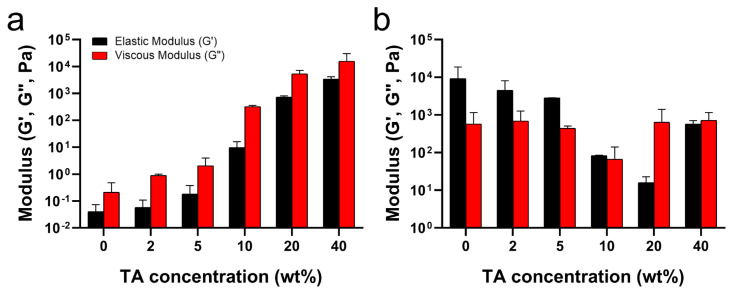
Average elastic (G′) and viscous (G″) moduli as a function of TA concentration at 4 (**a**) and 37 °C (**b**).

As mentioned above, the hydrogen bonds between TA and PluF in TA/PluF hydrogels significantly increased the G′ and G″ values at low temperatures (i.e., 4 °C) and suppressed the thermosensitive behaviors. As shown in Figure 4a, the G′ values at 4 °C were 0.06 ± 0.02 Pa, 0.09 ± 0.02 Pa, 0.3 ± 0.1 Pa, 14.2 ± 5.2 Pa, 0.8 ± 0.7 kPa, and 16.0 ± 6.9 kPa for PluF, 2TA/PluF, 5TA/PluF, 10TA/PluF, 20TA/PluF, and 40TA/PluF, respectively. Additionally, the G″ values (0.40 ± 0.02 Pa, 0.9 ± 0.8 Pa, 3.4 ± 0.7 Pa, 346.6 ± 303.1 Pa, 6.6 ± 4.0 kPa, and 25.8 ± 5.4 kPa for PluF, 2TA/PluF, 5TA/PluF, 10TA/PluF, 20TA/PluF, and 40TA/PluF, respectively) increased similarly to the G′ values in these hydrogels at 4 °C. Previously, tannic acid and polymer composite hydrogels demonstrated an increase in both elastic and viscous modulus values compared to those of polymer alone [46,47]. This enhancement is attributed to the hydrogen bonding between tannic acid and the polymers [46,47]. The observed increase in G′ and G″ values of TA/PluF composites at 4 °C might be due to the hydrogen bonding between TA and PluF. The G′ values were further monitored at 37 °C (Figure 4b) and decreased as a function of TA concentration until 20 wt% TA. However, the G′ values of 40TA/PluF hydrogels were 666.6 ± 485.7 Pa, much higher than those of 10TA/PluF (81.1 ± 84.3 Pa) and 20TA/PluF (20.8 ± 11.2 Pa) and lower than those of 2TA/PluF (7.0 ± 2.0 kPa) and 5TA/PluF (2.9 ± 2.9 kPa). These results suggest that increasing the TA concentrations in TA/PluF hydrogels could hinder the temperature sensitivity of Pluronic-based hydrogels via intermolecular hydrogen bonding between TA and Pluronic molecules.

### 2.3. In Vitro Stability of TA/PluF Hydrogels

**Figure 5:**
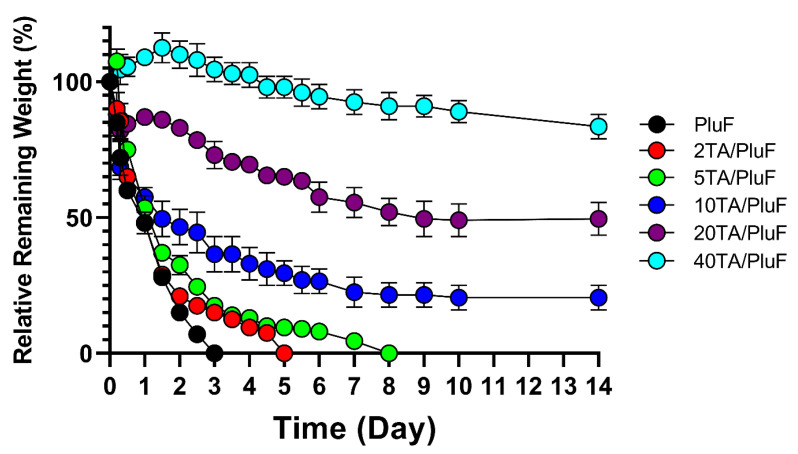
In vitro mass erosion rates of TA/PluF composites at different TA concentrations.

To monitor the differences in the in vitro stability of hydrogels, the relative remaining weights of the hydrogels were measured. As shown in Figure 5, PluF hydrogels alone were eroded within 3 days in the pH 7.4 PBS solution at 37 °C. As previously reported, PluF hydrogels rapidly erode under physiological conditions, restricting their versatile biomedical applications in drug delivery and tissue engineering [33]. Compared with the PluF hydrogels, the TA/PluF hydrogels showed enhanced in vitro stability as a function of TA concentration. The 2TA/PluF and 5TA/PluF hydrogels showed erosion profiles similar to those of the PluF hydrogels. However, the 10TA/PluF, 20TA/PluF, and 40TA/PluF hydrogels remained intact for at least 14 days. The relative remaining weights were 20.5 ± 4.5%, 49.0 ± 6.0, and 83.5 ± 4.5% for 10TA/PluF, 20TA/PluF, and 40TA/PluF after 14 days. These results are similar to those of previous reports, which indicated that PluF-based composite hydrogels have improved mechanical properties and stability owing to the incorporation of polymers (i.e., chitosan, hyaluronic acid, and alginate) [34,35,36,37]. Notably, TA/PluF composite hydrogels can be explored for various applications as effective drug delivery depots and long-lasting tissue engineering hydrogels by improving their stability.

### 2.4. Tissue Adhesiveness and Bursting Pressure of TA/PluF Hydrogels

**Figure 6:**
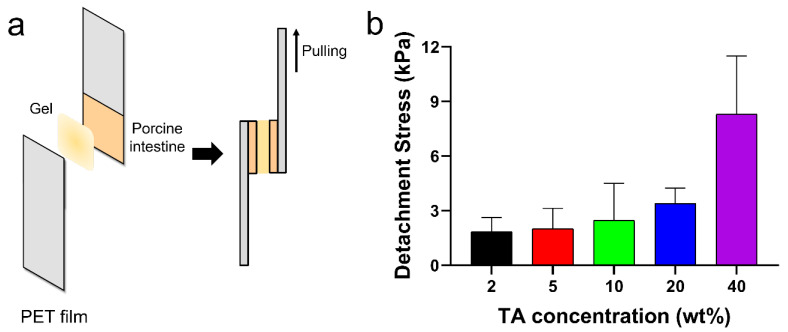
(**a**) Schematic illustrations of quantitative tissue adhesion analysis of TA/PluF composites. (**b**) Detachment stresses of TA/PluF composites with different TA concentrations.

The tissue adhesion forces of the TA/PluF composite hydrogels were measured using a universal testing machine. As shown in Figure 6a, fresh porcine intestinal tissues were attached to the PET films, and TA/PluF hydrogels were applied between the two intestinal tissues. The adhesive forces were measured by monitoring the tensile strength. The detachment stresses were 1.8 ± 0.6, 2.0 ± 0.8, 2.5 ± 1.7, 3.4 ± 0.6, and 8.3 ± 2.6 kPa for the 2TA/PluF, 5TA/PluF, 10TA/PluF, 20TA/PluF, and 40TA/PluF composites (Figure 6b). An increase in the TA concentration of the TA/PluF composites significantly affected the tissue adhesion properties of the hydrogels. Fibrin glues have been previously reported to show tissue adhesive strengths ranging from 0.5 to 10 kPa [48,49,50]. The adhesion forces of the 40TA/PluF composites were found to be comparable to those of fibrin glues.

**Figure 7:**
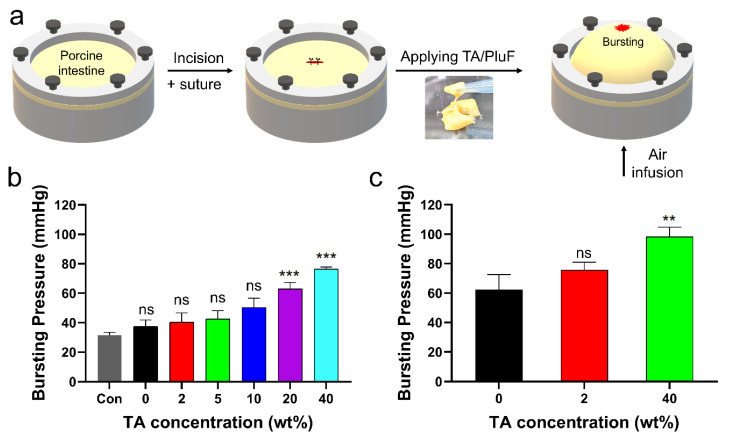
(**a**) Schematic illustrations of bursting pressure measurements. (**b**) Bursting pressures of porcine intestine with incision, after suturing (con), after suturing with PluF (0), and after suturing with TA/PluF as a function of TA concentration (2, 5, 10, 20, 40 wt%) at room temperature (*** *p* < 0.001). (**c**) Bursting pressures of porcine intestines with incision, after suturing with PluF without the TA, and after suturing with TA/PluF (2 and 40 wt% TA) at 37 °C (** *p* < 0.01). “ns” indicates not significant (*p* > 0.05).

The in vitro bursting pressures of porcine tissue with a 1 cm incision before and after applying the TA/PluF hydrogels were further monitored (Figure 7a). After preparing a length of 1 cm, single interrupted suturing was performed (Figure 7a, first and second). TA/PluF hydrogels of various compositions were then applied to the sutures. Air was blown into the porcine tissue, and the bursting pressure was monitored using a bursting pressure measurement system (Figure 7a, third). When a significant decrease in the detected pressures was observed, the pressures were considered the bursting pressures. The bursting pressures of the TA/PluF hydrogels were first measured at room temperature. As shown in Figure 7b, the bursting pressure of the normal porcine intestine after preparing suture holes was 31.6 ± 1.6 mmHg. This is similar to previous reports of the bursting pressures of normal porcine intestinal tissue after preparing suture holes (34.2 ± 1.6 mmHg) [51]. After preparing an incision and suturing by applying PluF hydrogels, the bursting pressures increased to 37.5 ± 3.5 mmHg. After applying TA/PluF hydrogels, the bursting pressures were increased to 40.2 ± 5.2, 42.4 ± 4.7, 50.4 ± 5.0, 63.0 ± 3.6, and 76.3 ± 1.3 mmHg for 2TA/PluF, 5TA/PluF, 10TA/PluF, 20TA/PluF, and 40TA/PluF composites, respectively. The bursting pressures of TA/PluF hydrogels were also measured at 37 °C (Figure 7c). The PluF hydrogels alone also showed slightly higher bursting pressures, which may have been due to the gelation of the hydrogels. The bursting pressures of the 2TA/PluF and 40TA/PluF hydrogels were increased to 75.8 ± 4.2 and 98.3 ± 5.4 mmHg, respectively. The increase in the TA concentration of the TA/PluF hydrogels significantly affected their bursting pressure. Thus, TA/PluF composite hydrogels are expected to be useful in various biomedical applications, particularly as tissue-sealing materials.

## 3. Conclusions

This study demonstrates temperature-sensitive sol–gel transition behaviors with the in vitro stability of tannic acid/Pluronic F127 (TA/PluF) composite hydrogels as a function of TA concentration. As the TA concentration in the TA/PluF hydrogels increased, the temperature-sensitive properties of the TA/PluF hydrogels were significantly suppressed by the intermolecular hydrogen bonds between TA and PluF. In addition, the elastic modulus (G′) values of the TA/PluF hydrogels increased as a function of TA concentration at 4 °C. In contrast, the G′ values of the hydrogels decreased from 0 to 20 wt% TA concentration in TA/PluF hydrogels at 37 °C. However, the G′ values increased at 40 wt% TA at 37 °C. Also, the in vitro stability and tissue adhesive properties of the TA/PluF hydrogels were improved by increasing the TA concentration. Furthermore, the bursting pressure in an in vitro incision model of porcine intestinal tissues was significantly enhanced by applying 40TA/PluF hydrogels. Our findings provide valuable insights into the design and development of effective and reliable hydrogel-based tissue adhesives for biomedical applications.

## 4. Materials and Methods

### 4.1. Materials

Tannic acid (TA) and Pluronic F-127 were purchased from Sigma-Aldrich (St. Louis, MO, USA). All the other chemicals were of analytical grade.

### 4.2. Preparation of Tannic Acid/Pluronic F127 Composite Hydrogels (TA/PluF Hydrogels)

To prepare the TA/PluF hydrogels, TA and PluF solutions were mixed at 4 °C and room temperature at predetermined concentrations. Briefly, TA and PluF were dissolved in a pH 7.4 PBS solution at room temperature and 4 °C, respectively. After the complete dissolution of TA and PluF, the two solutions were repeatedly mixed at 4 °C and room temperature. After the homogeneous mixing of the TA and PluF solutions, all measurements were performed. Equal volumes of the two solutions with 2X-concentrated polymer solutions were mixed to obtain the final concentrations. 2TA/PluF hydrogels (2 wt% TA and 18 wt% PluF) were prepared by mixing 4 wt% TA and 36 wt% PluF in equal volumes. To monitor the temperature-responsive behavior and rheological properties of the TA/PluF hydrogels, the TA concentrations were varied from 2 to 40 wt% (2, 5, 10, 20, and 40 wt%). Additionally, the PluF concentration was varied from 14 to 28 wt% (14, 16, 18, 20, 22, 24, 26, and 28 wt%). To evaluate the in vitro mass erosion behavior, tissue adhesive properties, and bursting pressures, the concentrations of TA were varied as described above. However, the PluF concentration was fixed at 18 wt% to confirm the effects of TA concentration in the TA/PluF hydrogels.

### 4.3. Sol–Gel Transition Behaviors of TA/PluF Hydrogels

To obtain the sol–gel transition curves of TA/PluF hydrogels, a vial-inverting test with temperature changes between 4 and 100 °C was performed using a refrigerated/heating circulator (JEIO Tech, Daejeon, Republic of Korea) equipped with a water bath. Briefly, TA/PluF solutions (500 μL) with various concentrations were placed in 2 mL test tubes, which were then placed in the water bath of the refrigerated/heating circulator at 4 °C and stabilized for 5 min. The temperature of the water bath was gradually increased at a rate of 1 °C per 5 min. The sol–gel phase transition temperature of the TA/PluF hydrogels was monitored when no flow was observed for 5 min after inverting the test tubes. PluF hydrogels with and without TA were used as controls. All samples were analyzed in triplicate.

### 4.4. Rheological Studies of TA/PluF Hydrogels

Rheological properties were evaluated using a rotating rheometer (Kinexus Lab+, Netzsch, Germany) equipped with a parallel-plate geometry (diameter: 20 mm). Temperature sweep measurements were performed to monitor the gelation temperature and thermosensitive behavior of the TA/PluF hydrogels. TA/PluF mixtures (150 μL) were placed on the rheometer plate. The elastic modulus (solid-like property, G′) and viscous modulus (liquid-like property, G″) values of the TA/PluF hydrogels were monitored as a function of temperature from 4 to 60 °C. After confirming the linear viscoelastic region, the frequency was fixed at 1 Hz with a constant strain of 1% during the temperature sweep measurements. The crossover points of G′ and G″ were considered as gelation temperatures. Additionally, the G′ values of the TA/PluF hydrogels were collected at both 4 and 60 °C to verify the modulus change as a function of the TA concentration. Furthermore, the G′ and G″ values of TA/PluF hydrogels with thermal cycles of heating (37 °C) and cooling (4 °C) were monitored. PluF hydrogels alone were used as the controls. All measurements were performed in triplicate.

### 4.5. In Vitro Mass Erosion Behavior of TA/PluF Hydrogels

To measure the in vitro mass erosion behavior of the TA/PluF hydrogels, their relative remaining weights in PBS solution (pH 7.4) were measured at predetermined time intervals. Briefly, TA/PluF hydrogels (0.5 mL) were placed in 2 mL test tubes and stabilized at 37 °C for 30 min. Then, a pH 7.4 PBS solution (1 mL) at 37 °C was added to the TA/PluF hydrogel mass. The hydrogels were incubated at 37 °C under mild agitation during the in vitro stability tests. The supernatants from the test tubes were removed and the weights of the remaining hydrogels were measured at predetermined time intervals. The remaining weights of the solid hydrogels were compared with the initial hydrogel weights to determine fractional mass loss. PluF hydrogels without TA were used as controls. All samples were analyzed in triplicate.

### 4.6. Tissue Adhesion of TA/PluF Hydrogels

To measure the tissue adhesive properties, the detachment stresses of the TA/PluF hydrogels from porcine intestinal tissues were measured using a universal testing machine (UTM, Instron 5583, Instron, Norwood, MA, USA) with a load cell of 50 N. Briefly, the TA/PluF hydrogels were freshly prepared before the adhesive force measurements. The concentrations ranged from 2 to 40 wt% (2, 5, 10, 20, and 40 wt%) for TA and were fixed at 18 wt% for PluF. Fresh porcine intestinal tissues (Bucknam Butcher Shop, Iksan, Republic of Korea) were cut into 1 × 1 cm^2^ squares and attached to the edges of polyethylene terephthalate (PET) films (1 × 3 cm^2^). Then, two intestinal tissue samples were overlapped on the PET films by 1 × 1 cm^2^, and hydrogels were applied between two tissues. The tensile strength of TA/PluF hydrogels was measured by pulling the UTM probe at a crosshead speed of 1 mm/min. PluF hydrogels without TA were used as controls. All measurements were performed in triplicate.

### 4.7. In Vitro Bursting Pressure Measurements

To measure the sealing effects of the TA/PluF hydrogels, the in vitro bursting pressures of porcine intestine samples with an incision before and after applying the TA/PluF hydrogels were measured using bursting pressure measurement systems. Briefly, fresh intestines (Bucknam Butcher Shop, Iksan, Korea) were fixed in plastic containers connected to indicators, pressure transmitters, and recorders. The center of the fresh intestine was cut to a length of 1 cm using scissors, and TA/PluF hydrogels were applied to the incision sites. Air was then blown into the plastic containers and the bursting pressure was monitored. The bursting pressure was measured when a significant decrease in pressure was observed due to air leakage from the intestine. All measurements were performed in triplicate.

### 4.8. Statistical Analysis

To test the normality of samples, the Shapiro–Wilk test (α = 0.05, *n* = 3) was performed using Prism 8.1.0 software (GraphPad Software Inc., La Jolla, CA, USA). Statistical significance was analyzed using a one-way analysis of variance (ANOVA) with Tukey’s test for multiple comparisons. Significance levels were assigned as follows: * *p* < 0.05, ** *p* < 0.01, and *** *p* < 0.001.

## Data Availability

The data presented in this study are available on request from the corresponding author.
